# 4-(4-Fluoro­phen­yl)-1-methoxy­methyl-2-phenyl-1*H*-imidazole

**DOI:** 10.1107/S1600536809042366

**Published:** 2009-10-23

**Authors:** Roland Selig, Dieter Schollmeyer, Wolfgang Albrecht, Stefan Laufer

**Affiliations:** a Eberhard-Karls-University Tübingen, Auf der Morgenstelle 8, D-72076 Tübingen, Germany; b University Mainz, Duesbergweg 10-14, D-55099 Mainz, Germany; c c-a-i-r biosciences GmbH, Paul-Ehrlich-Strasse 15, 72076 Tübingen, Germany

## Abstract

In the crystal structure of the title compound, C_17_H_15_FN_2_O, the mol­ecules form a three-dimensional network stabilized by π–π inter­actions between two imidazole rings related by a centre of symmetry. The distance between the centroids is 3.5488 (8) Å. The imidazole ring makes dihedral angles of 14.30 (7) and 33.39 (7)° with the 4-fluoro­phenyl ring and the phenyl ring, respectively.

## Related literature

For the preparation of diaryl­imidazoles, see: Li *et al.* (2002 ▶). For synthesis of and with related diarylimidazoles, see: Liverton *et al.* (1999 ▶); Kawasaki *et al.* (1996 ▶).
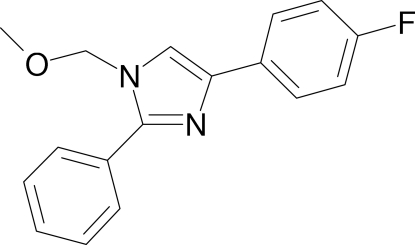


## Experimental

### 

#### Crystal data

C_17_H_15_FN_2_O
*M* *_r_* = 282.31
Monoclinic, 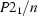

*a* = 10.524 (1) Å
*b* = 11.248 (1) Å
*c* = 11.981 (1) Å
β = 92.206 (3)°
*V* = 1417.2 (2) Å^3^
*Z* = 4
Mo *K*α radiation
μ = 0.09 mm^−1^
*T* = 173 K
0.40 × 0.30 × 0.10 mm

#### Data collection

Bruker SMART CCD diffractometer
Absorption correction: none
37183 measured reflections
3319 independent reflections
2745 reflections with *I* > 2σ(*I*)
*R*_int_ = 0.043

#### Refinement

*R*[*F* ^2^ > 2σ(*F* ^2^)] = 0.037
*wR*(*F* ^2^) = 0.106
*S* = 1.07
3319 reflections
191 parameters
H-atom parameters constrained
Δρ_max_ = 0.28 e Å^−3^
Δρ_min_ = −0.18 e Å^−3^

### 

Data collection: *APEX2* (Bruker, 2006 ▶); cell refinement: *SAINT* (Bruker, 2006 ▶); data reduction: *SAINT*; program(s) used to solve structure: *SIR97* (Altomare *et al.*, 1999 ▶); program(s) used to refine structure: *SHELXL97* (Sheldrick, 2008 ▶); molecular graphics: *PLATON* (Spek, 2009 ▶); software used to prepare material for publication: *PLATON*.

## Supplementary Material

Crystal structure: contains datablocks I, global. DOI: 10.1107/S1600536809042366/bt5098sup1.cif

Structure factors: contains datablocks I. DOI: 10.1107/S1600536809042366/bt5098Isup2.hkl

Additional supplementary materials:  crystallographic information; 3D view; checkCIF report

## Figures and Tables

**Fig. 1.**
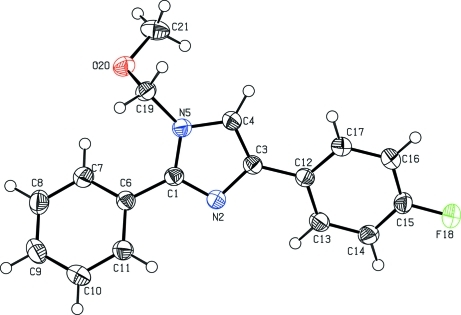
View of compound I. Displacement ellipsoids are drawn at the 50% probability level.

## supplementary crystallographic information

### Comment

Metallated imidazole intermediates can be widely used in the preparation of imidazole derivatives. To avoid N-metallation, N-protection of the imidazole is necessary. Our approach was a direct regioselective N-protection of the diarylimidazole. The regioselectivity is controlled by the steric effect of the aryl substituent in C-4 position. The title compound forms a three dimensional network stabilized by π-π interactions between two symmetry related imidazole nuclei. The distance between the centroids is 3.5488 (8) Å. The imidazole ring makes dihedral angles of 14.30 (7)° and 33.39 (7)° to the 4-fluorophenyl ring and the phenyl ring, respectively.

### Experimental

4-(4-fluorophenyl)-2-phenyl-1*H*-imidazole (3 g 13 mmol) was dissolved in dry THF (30 ml). After cooling to 273 K sodium bis(trimetyl silyl)amide (8.2 ml, 16 mmol) was added dropwise. The reaction mixture was stirred for 30 min at this temperature. Methoxymethylchloride (2.1*M* in toluene) (12 ml, 25 mmol) was added dropwise. Stirring was continued at 273 K for 30 min and further 60 min at room temperature. Then concentrated aqueous ammonium chloride solution (30 ml) was added, the reaction mixture was stirred for 1 h. After extraction with ethyl acetate the organic layer was washed twice with water, dried over sodium sulfate and evaporated under reduced pressure. The crude product was purified by flash chromatography to yield 4-(4-fluorophenyl)-1-(methoxymethyl)-2-phenyl-1*H*-imidazole (42%).

### Refinement

Hydrogen atoms   were placed at calculated positions with C—H = 0.95 Å (aromatic) or 0.98–0.99 Å (*sp*^3^ C-atom) and refined in the riding-model approximation with isotropic displacement parameters (set at 1.2–1.5 times of the *U*_eq_ of the parent atom).

### Crystal data

**Table:**

C_17_H_15_FN_2_O	*F*(000) = 592
*M**_r_* = 282.31	*D*_x_ = 1.323 Mg m^−^^3^
Monoclinic, *P*2_1_/*n*	Mo *K*α radiation, λ = 0.71069 Å
Hall symbol: -P 2yn	Cell parameters from 9878 reflections
*a* = 10.524 (1) Å	θ = 2.4–27.6°
*b* = 11.2480 (11) Å	µ = 0.09 mm^−^^1^
*c* = 11.9810 (12) Å	*T* = 173 K
β = 92.206 (3)°	Needle, colourless
*V* = 1417.2 (2) Å^3^	0.40 × 0.30 × 0.10 mm
*Z* = 4	

### Data collection

**Table:**

Bruker SMART CCD diffractometer	2745 reflections with *I* > 2σ(*I*)
Radiation source: sealed Tube	*R*_int_ = 0.043
graphite	θ_max_ = 27.7°, θ_min_ = 2.5°
CCD scan	*h* = −13→13
37183 measured reflections	*k* = −14→14
3319 independent reflections	*l* = −15→15

### Refinement

**Table:**

Refinement on *F*^2^	Primary atom site location: structure-invariant direct methods
Least-squares matrix: full	Secondary atom site location: difference Fourier map
*R*[*F*^2^ > 2σ(*F*^2^)] = 0.037	Hydrogen site location: inferred from neighbouring sites
*wR*(*F*^2^) = 0.106	H-atom parameters constrained
*S* = 1.07	*w* = 1/[σ^2^(*F*_o_^2^) + (0.0415*P*)^2^ + 0.5423*P*] where *P* = (*F*_o_^2^ + 2*F*_c_^2^)/3
3319 reflections	(Δ/σ)_max_ = 0.001
191 parameters	Δρ_max_ = 0.28 e Å^−^^3^
0 restraints	Δρ_min_ = −0.17 e Å^−^^3^

### Special details

**Table:**

Geometry. All e.s.d.'s (except the e.s.d. in the dihedral angle between two l.s. planes) are estimated using the full covariance matrix. The cell e.s.d.'s are taken into account individually in the estimation of e.s.d.'s in distances, angles and torsion angles; correlations between e.s.d.'s in cell parameters are only used when they are defined by crystal symmetry. An approximate (isotropic) treatment of cell e.s.d.'s is used for estimating e.s.d.'s involving l.s. planes.
Refinement. Refinement of *F*^2^ against ALL reflections. The weighted *R*-factor *wR* and goodness of fit *S* are based on *F*^2^, conventional *R*-factors *R* are based on *F*, with *F* set to zero for negative *F*^2^. The threshold expression of *F*^2^ > σ(*F*^2^) is used only for calculating *R*-factors(gt) *etc*. and is not relevant to the choice of reflections for refinement. *R*-factors based on *F*^2^ are statistically about twice as large as those based on *F*, and *R*- factors based on ALL data will be even larger.

### Fractional atomic coordinates and isotropic or equivalent isotropic displacement parameters (Å^2^)

**Table:**

	*x*	*y*	*z*	*U*_iso_*/*U*_eq_	
C1	0.37469 (12)	0.48140 (11)	0.36743 (10)	0.0272 (3)	
N2	0.47445 (10)	0.45452 (9)	0.30842 (8)	0.0284 (2)	
C3	0.55709 (12)	0.54982 (10)	0.32055 (10)	0.0268 (3)	
C4	0.50522 (12)	0.63404 (11)	0.38760 (10)	0.0289 (3)	
H4	0.5423	0.7079	0.4093	0.035*	
N5	0.38931 (10)	0.59103 (9)	0.41746 (8)	0.0281 (2)	
C6	0.26578 (12)	0.40118 (11)	0.38101 (10)	0.0282 (3)	
C7	0.14177 (12)	0.44168 (12)	0.39029 (11)	0.0348 (3)	
H7	0.1246	0.5246	0.3893	0.042*	
C8	0.04297 (14)	0.36122 (14)	0.40103 (13)	0.0414 (3)	
H8	−0.0413	0.3896	0.4082	0.050*	
C9	0.06605 (14)	0.23974 (14)	0.40139 (12)	0.0424 (3)	
H9	−0.0019	0.1852	0.4097	0.051*	
C10	0.18894 (14)	0.19848 (13)	0.38951 (12)	0.0390 (3)	
H10	0.2051	0.1154	0.3879	0.047*	
C11	0.28813 (13)	0.27826 (11)	0.37999 (11)	0.0323 (3)	
H11	0.3723	0.2494	0.3727	0.039*	
C12	0.68112 (12)	0.55033 (10)	0.26877 (10)	0.0270 (3)	
C13	0.70870 (13)	0.46715 (11)	0.18610 (11)	0.0318 (3)	
H13	0.6454	0.4118	0.1618	0.038*	
C14	0.82693 (13)	0.46451 (11)	0.13935 (11)	0.0342 (3)	
H14	0.8453	0.4076	0.0837	0.041*	
C15	0.91715 (13)	0.54576 (12)	0.17496 (11)	0.0334 (3)	
C16	0.89444 (13)	0.62991 (12)	0.25570 (12)	0.0370 (3)	
H16	0.9582	0.6856	0.2784	0.044*	
C17	0.77620 (13)	0.63091 (12)	0.30265 (11)	0.0340 (3)	
H17	0.7593	0.6875	0.3590	0.041*	
F18	1.03312 (8)	0.54292 (8)	0.12881 (8)	0.0482 (2)	
C19	0.30688 (12)	0.64839 (12)	0.49708 (10)	0.0312 (3)	
H19A	0.2692	0.5866	0.5445	0.037*	
H19B	0.3591	0.7012	0.5464	0.037*	
O20	0.20865 (9)	0.71516 (8)	0.44560 (8)	0.0363 (2)	
C21	0.25020 (16)	0.82639 (13)	0.40291 (12)	0.0446 (4)	
H21A	0.2889	0.8737	0.4639	0.067*	
H21B	0.1772	0.8694	0.3694	0.067*	
H21C	0.3130	0.8125	0.3460	0.067*	

### Atomic displacement parameters (Å^2^)

**Table:**

	*U*^11^	*U*^22^	*U*^33^	*U*^12^	*U*^13^	*U*^23^
C1	0.0285 (6)	0.0255 (6)	0.0277 (6)	0.0024 (5)	0.0004 (4)	0.0019 (4)
N2	0.0283 (5)	0.0269 (5)	0.0302 (5)	0.0001 (4)	0.0024 (4)	0.0008 (4)
C3	0.0284 (6)	0.0253 (6)	0.0265 (6)	0.0001 (5)	−0.0001 (4)	0.0030 (4)
C4	0.0293 (6)	0.0260 (6)	0.0315 (6)	−0.0014 (5)	0.0017 (5)	0.0010 (5)
N5	0.0294 (5)	0.0259 (5)	0.0291 (5)	0.0012 (4)	0.0026 (4)	−0.0001 (4)
C6	0.0287 (6)	0.0290 (6)	0.0269 (6)	−0.0012 (5)	0.0016 (4)	−0.0001 (5)
C7	0.0311 (7)	0.0337 (7)	0.0394 (7)	0.0012 (5)	0.0013 (5)	−0.0020 (5)
C8	0.0294 (7)	0.0493 (9)	0.0458 (8)	−0.0034 (6)	0.0047 (6)	−0.0048 (6)
C9	0.0398 (8)	0.0453 (8)	0.0424 (8)	−0.0156 (6)	0.0044 (6)	−0.0012 (6)
C10	0.0477 (8)	0.0295 (7)	0.0395 (7)	−0.0060 (6)	0.0000 (6)	0.0004 (5)
C11	0.0328 (7)	0.0294 (6)	0.0346 (6)	0.0004 (5)	0.0008 (5)	−0.0005 (5)
C12	0.0291 (6)	0.0245 (6)	0.0275 (6)	0.0009 (5)	0.0019 (4)	0.0047 (4)
C13	0.0350 (7)	0.0277 (6)	0.0327 (6)	−0.0038 (5)	0.0029 (5)	−0.0005 (5)
C14	0.0398 (7)	0.0288 (6)	0.0345 (6)	0.0000 (5)	0.0095 (5)	−0.0013 (5)
C15	0.0302 (7)	0.0327 (7)	0.0380 (7)	0.0012 (5)	0.0092 (5)	0.0053 (5)
C16	0.0340 (7)	0.0326 (7)	0.0447 (8)	−0.0070 (5)	0.0056 (6)	−0.0033 (6)
C17	0.0351 (7)	0.0295 (6)	0.0377 (7)	−0.0020 (5)	0.0058 (5)	−0.0043 (5)
F18	0.0380 (5)	0.0473 (5)	0.0609 (6)	−0.0051 (4)	0.0216 (4)	−0.0058 (4)
C19	0.0341 (7)	0.0293 (6)	0.0305 (6)	0.0043 (5)	0.0060 (5)	0.0008 (5)
O20	0.0355 (5)	0.0292 (5)	0.0444 (5)	0.0070 (4)	0.0049 (4)	0.0032 (4)
C21	0.0661 (10)	0.0279 (7)	0.0394 (7)	0.0026 (7)	−0.0033 (7)	0.0026 (6)

### Geometric parameters (Å, °)

**Table:**

C1—N2	1.3231 (15)	C11—H11	0.9500
C1—N5	1.3772 (16)	C12—C17	1.3987 (18)
C1—C6	1.4727 (17)	C12—C13	1.4010 (17)
N2—C3	1.3844 (15)	C13—C14	1.3844 (18)
C3—C4	1.3691 (17)	C13—H13	0.9500
C3—C12	1.4666 (17)	C14—C15	1.3738 (19)
C4—N5	1.3725 (16)	C14—H14	0.9500
C4—H4	0.9500	C15—F18	1.3595 (15)
N5—C19	1.4637 (15)	C15—C16	1.3808 (19)
C6—C7	1.3907 (18)	C16—C17	1.3853 (18)
C6—C11	1.4026 (18)	C16—H16	0.9500
C7—C8	1.3882 (19)	C17—H17	0.9500
C7—H7	0.9500	C19—O20	1.4014 (15)
C8—C9	1.388 (2)	C19—H19A	0.9900
C8—H8	0.9500	C19—H19B	0.9900
C9—C10	1.387 (2)	O20—C21	1.4266 (17)
C9—H9	0.9500	C21—H21A	0.9800
C10—C11	1.3846 (19)	C21—H21B	0.9800
C10—H10	0.9500	C21—H21C	0.9800
			
N2—C1—N5	111.07 (11)	C17—C12—C13	118.20 (12)
N2—C1—C6	123.78 (11)	C17—C12—C3	121.21 (11)
N5—C1—C6	125.09 (11)	C13—C12—C3	120.56 (11)
C1—N2—C3	105.97 (10)	C14—C13—C12	120.95 (12)
C4—C3—N2	109.53 (11)	C14—C13—H13	119.5
C4—C3—C12	128.82 (11)	C12—C13—H13	119.5
N2—C3—C12	121.62 (11)	C15—C14—C13	118.77 (12)
C3—C4—N5	106.71 (11)	C15—C14—H14	120.6
C3—C4—H4	126.6	C13—C14—H14	120.6
N5—C4—H4	126.6	F18—C15—C14	118.59 (12)
C4—N5—C1	106.72 (10)	F18—C15—C16	118.90 (12)
C4—N5—C19	124.72 (11)	C14—C15—C16	122.51 (12)
C1—N5—C19	128.27 (11)	C15—C16—C17	118.19 (12)
C7—C6—C11	118.81 (12)	C15—C16—H16	120.9
C7—C6—C1	123.02 (12)	C17—C16—H16	120.9
C11—C6—C1	118.13 (11)	C16—C17—C12	121.38 (12)
C8—C7—C6	120.16 (13)	C16—C17—H17	119.3
C8—C7—H7	119.9	C12—C17—H17	119.3
C6—C7—H7	119.9	O20—C19—N5	113.27 (10)
C9—C8—C7	120.70 (14)	O20—C19—H19A	108.9
C9—C8—H8	119.7	N5—C19—H19A	108.9
C7—C8—H8	119.7	O20—C19—H19B	108.9
C10—C9—C8	119.54 (13)	N5—C19—H19B	108.9
C10—C9—H9	120.2	H19A—C19—H19B	107.7
C8—C9—H9	120.2	C19—O20—C21	113.37 (11)
C11—C10—C9	120.04 (13)	O20—C21—H21A	109.5
C11—C10—H10	120.0	O20—C21—H21B	109.5
C9—C10—H10	120.0	H21A—C21—H21B	109.5
C10—C11—C6	120.72 (13)	O20—C21—H21C	109.5
C10—C11—H11	119.6	H21A—C21—H21C	109.5
C6—C11—H11	119.6	H21B—C21—H21C	109.5
			
N5—C1—N2—C3	0.12 (13)	C9—C10—C11—C6	−0.7 (2)
C6—C1—N2—C3	−177.34 (11)	C7—C6—C11—C10	−0.84 (19)
C1—N2—C3—C4	−0.04 (13)	C1—C6—C11—C10	−178.34 (12)
C1—N2—C3—C12	178.28 (10)	C4—C3—C12—C17	13.98 (19)
N2—C3—C4—N5	−0.04 (13)	N2—C3—C12—C17	−163.99 (11)
C12—C3—C4—N5	−178.21 (11)	C4—C3—C12—C13	−168.03 (12)
C3—C4—N5—C1	0.11 (13)	N2—C3—C12—C13	14.00 (17)
C3—C4—N5—C19	174.26 (11)	C17—C12—C13—C14	0.16 (19)
N2—C1—N5—C4	−0.15 (13)	C3—C12—C13—C14	−177.89 (11)
C6—C1—N5—C4	177.27 (11)	C12—C13—C14—C15	−0.4 (2)
N2—C1—N5—C19	−174.02 (11)	C13—C14—C15—F18	−179.97 (12)
C6—C1—N5—C19	3.40 (19)	C13—C14—C15—C16	0.1 (2)
N2—C1—C6—C7	−146.46 (13)	F18—C15—C16—C17	−179.40 (12)
N5—C1—C6—C7	36.44 (18)	C14—C15—C16—C17	0.6 (2)
N2—C1—C6—C11	30.93 (17)	C15—C16—C17—C12	−0.8 (2)
N5—C1—C6—C11	−146.17 (12)	C13—C12—C17—C16	0.49 (19)
C11—C6—C7—C8	1.54 (19)	C3—C12—C17—C16	178.53 (12)
C1—C6—C7—C8	178.91 (12)	C4—N5—C19—O20	99.09 (14)
C6—C7—C8—C9	−0.7 (2)	C1—N5—C19—O20	−88.05 (15)
C7—C8—C9—C10	−0.8 (2)	N5—C19—O20—C21	−76.19 (14)
C8—C9—C10—C11	1.5 (2)		
